# Respiration by “marine snow” at high hydrostatic pressure: Insights from continuous oxygen measurements in a rotating pressure tank

**DOI:** 10.1002/lno.11791

**Published:** 2021-05-14

**Authors:** Peter Stief, Marcus Elvert, Ronnie N. Glud

**Affiliations:** ^1^ HADAL & Nordcee, Department of Biology University of Southern Denmark Odense Denmark; ^2^ MARUM—Center for Marine Environmental Sciences University of Bremen Bremen Germany; ^3^ Faculty of Geosciences University of Bremen Bremen Germany; ^4^ Danish Institute for Advanced Study (DIAS) University of Southern Denmark Odense Denmark; ^5^ Department of Ocean and Environmental Sciences Tokyo University of Marine Science and Technology Tokyo Japan

## Abstract

It is generally anticipated that particulate organic carbon (POC) for most part is degraded by attached microorganisms during the descent of “marine snow” aggregates toward the deep sea. There is, however, increasing evidence that fresh aggregates can reach great depth and sustain relatively high biological activity in the deep sea. Using a novel high‐pressure setup, we tested the hypothesis that increasing levels of hydrostatic pressure inhibit POC degradation in aggregates rapidly sinking to the ocean interior. Respiration activity, a proxy for POC degradation, was measured directly and continuously at up to 100 MPa (corresponding to 10 km water depth) in a rotating pressure tank that keeps the aggregates in a sinking mode. Model diatom‐bacteria aggregates, cultures of the aggregate‐forming diatom *Skeletonema marinoi*, and seawater microbial communities devoid of diatoms showed incomplete and complete inhibition of respiration activity when exposed to pressure levels of 10–50 and 60–100 MPa, respectively. This implies reduced POC degradation and hence enhanced POC export to hadal trenches through fast‐sinking, pressure‐exposed aggregates. Notably, *continuous* respiration measurements at ≥50 MPa revealed curved instead of linear oxygen time series whenever *S. marinoi* was present, which was not captured by *discrete* respiration measurements. These curvatures correspond to alternating phases of high and low respiration activity likely connected to pressure effects on unidentified metabolic processes in *S. marinoi*.

Phototrophic primary production in the ocean is limited to sunlit surface waters where much of the organic carbon produced is also recycled (Martin et al. 1987; Buesseler et al. 2007). Only a small fraction of primary production is exported to greater water depth as particulate organic carbon (POC), which drives the oceanic “biological carbon pump” (BCP) (Berger et al. 1988; Jahnke 1996; Turner 2015). Major components of the BCP include sinking phytodetritus aggregates, fecal pellets, and zooplankton carcasses, which collectively constitute “marine snow” (Simon et al. 2002; Thornton 2002; Turner 2015). The BCP supplies deep‐sea food webs with food and energy, but also leads to carbon sequestration in the seafloor (Lochte and Turley 1988; Turley et al. 1995; Buesseler et al. 2007). The vertical POC flux is strongly attenuated in the mesopelagic zone due to microbial organic matter degradation and grazing, which decreases the transfer efficiency of the BCP as expressed by the so‐called Martin curve (Martin et al. 1987; Buesseler et al. 2007; Belcher et al. 2016; Cram et al. 2018).

Two common observations question the general validity of the “Martin curve.” First, the vertical POC flux determined with sediment traps is often lower than the deep‐sea carbon demand (Martin et al. 1987; Aristegui et al. 2009; Herndl and Reinthaler 2013). Second, there are surprising records of relatively fresh organic matter and viable phytoplankton very deep in the ocean (Lochte and Turley 1988; Smith et al. 1996; Danovaro et al. 2003; Boetius et al. 2013; Glud et al. 2013; Agusti et al. 2015; Thomsen et al. 2017; Glud et al. 2021). Several mechanisms have been proposed as to why the attenuation of the vertical POC flux is weaker than predicted by the “Martin curve,” such as (1) the occurrence of nonsinking particles that are not readily collected by sediment traps (Martin et al. 1987; Herndl and Reinthaler 2013; Belcher et al. 2016), (2) the existence of “particle injection pumps” that supplement the gravitational sinking of particles (Boyd et al. 2019), (3) the significance of primary production through chemosynthesis in the aphotic zone (Aristegui et al. 2009; Tamburini et al. 2009; Herndl and Reinthaler 2013), and (4) the inhibitory effect of hydrostatic pressure on organic carbon degradation in sinking particles (Turley 1993; Tamburini et al. 2006; de Jesus Mendes et al. 2007).

Hydrostatic pressure is increasingly recognized as an important environmental parameter that controls microbial metabolic activity and thus biogeochemical element cycling in the ocean (Picard and Daniel 2013; Tamburini et al. 2013). Both the decompression of deep‐sea microbial communities (Tholosan et al. 1999; Tamburini et al. 2003; Wannicke et al. 2015; Garel et al. 2019) and the compression of surface‐water microbial communities (Turley 1993; Tholosan et al. 1999; Tamburini et al. 2006; de Jesus Mendes et al. 2007) often entail a decrease in metabolic activity. Microorganisms associated with sinking aggregates experience rapid increases in pressure level of 1–7 MPa/d (Alldredge and Gotschalk 1989; Agusti et al. 2015) or >20 MPa/d (de Jesus Mendes et al. 2007), which may significantly inhibit their metabolic activity.

Microbial degradation of sinking aggregates can be quantified by measuring oxygen consumption rates. The main technical challenges of oxygen measurements on sinking aggregates under high‐pressure conditions are that aggregates need to be kept in a sinking mode and oxygen should be measured directly and continuously to minimize artifacts caused by operational pressure changes. We therefore devised a cylindrical pressure tank that can be rotated along its long axis and allows optode‐based, *continuous* oxygen measurements at pressure levels of up to 100 MPa (corresponding to 10 km water depth). This experimental setup was used to test the hypothesis that increasing pressure levels inhibit microbial respiration activity and thus organic carbon degradation in sinking aggregates. The effect of high hydrostatic pressure was separately unraveled for (1) model diatom‐bacteria aggregates, (2) axenic cultures of the aggregate‐forming diatom *Skeletonema marinoi*, and (3) seawater microbial communities devoid of diatoms. By experimentally increasing the pressure level, the rapid sinking of these microorganisms and their associations to greater water depth was simulated. By comparing pressure and control incubations, the effect of high pressure levels was quantified for each corresponding sample type and water depth. The observed depth‐dependent inhibition pattern was used to assess organic carbon preservation in pressure‐exposed model aggregates, which has implications for the natural POC export to hadal trenches.

## Methods

### Sample preparation

#### Nutrient‐enriched seawater

Natural surface seawater was enriched with spent diatom growth media (L1) and dissolved inorganic nitrogen (DIN) and phosphorus (DIP) to favor the growth and activity of microorganisms that would likely thrive also inside diatom‐bacteria aggregates. Spent diatom growth media are replete in dissolved organic carbon (DOC) from diatom exudates (which are likely also present in diatom‐bacteria aggregates), but are depleted in inorganic nutrients and therefore DIN and DIP were supplemented. It was intended that the enriched microbial communities displayed higher respiration rates than natural microbial communities in surface and deep waters of the ocean.

Seawater (salinity 33) was collected in the Kattegat off Helsingør at 28 m water depth. Upon arrival in the laboratory, 60 L of seawater were 5‐*μ*m‐filtered and stored at 3°C and in darkness to keep microalgae abundance at a minimum. Since the *S. marinoi* strain used in this study was adapted to salinity 30, the natural seawater that was designated for aggregate production was adjusted to salinity 30 by adding deionized water. Cell‐free, spent L1 media were obtained from *S. marinoi* cultures.

Over the course of 12 months of experimenting, four independent 5‐L batches of nutrient‐enriched seawater were produced by amending 4.5–4.8 L of stored seawater with 0.2–0.5 L of spent L1 media. Based on initial DOC, NO_3_^−^, NH_4_^+^, and PO_4_^3−^ concentrations in this mixture (Table S1), different aliquots of NH_4_Cl, and Na_2_HPO_4_ stock solutions were added to arrive at a C:N:P ratio of 106:16:1 in the nutrient‐enriched seawater. Each batch of nutrient‐enriched seawater was matured at 3°C and in darkness for 1–2 weeks and then used for pressure experiments for up to 4 weeks before a new batch was produced. One day before each pressure experiment, 240 mL of matured, nutrient‐enriched seawater was amended with 10 mL of cell‐free, spent L1 media to avoid organic‐carbon limitation during the experiment.

#### Skeletonema marinoi culture

An axenic strain of the marine, chain‐forming diatom *Skeletonema marinoi* (CCMP1332) was obtained from NCMA (Bigelow) and cultured in L1 medium (Guillard and Hargraves 1993) prepared with 0.22‐*μ*m‐filtered and autoclaved seawater collected in the Kattegat and adjusted to salinity 30. The cultivation temperature was 14°C and the light: dark cycle was 14:10 h. Stationary‐phase *S. marinoi* cultures were used for pressure experiments and for aggregate production. Axenia of the *S. marinoi* cultures was ascertained by both phase‐contrast microscopy and phospholipid fatty acid (PLFA) analysis targeting bacteria‐specific (iC_15:0_) and diatom‐specific fatty acids (C_20:5_). The abundance of the bacterial branched FA iC_15:0_ relative to the diatom‐specific polyunsaturated FA C_20:5_ in the *S. marinoi* culture used for pressure experiments was 0.05% (Table S2) and specifically, absolute amounts of iC_15:0_ were indistinguishable from laboratory blank runs.

#### Diatom‐bacteria aggregates

Using *S. marinoi* cultures, natural seawater, and a plankton wheel, a new batch of diatom‐bacteria aggregates was produced for each pressure experiment. Stationary‐phase *S. marinoi* cultures (50–100 mL) were mixed with stored seawater (500–550 mL) and filled into glass bottles (600 mL). These aggregate production bottles were mounted on a plankton wheel and continuously rotated to keep the diatom cells and later on the aggregates in suspension. Within 1–5 d, spherical‐to‐ellipsoidal aggregates formed with major axis lengths of 3–5 mm. The rotation speed of the plankton wheel was adjusted every day to minimize the risk of aggregates colliding with the walls of the bottle. Pressure experiments were made with 4–8 d old aggregates.

### Rotating incubation tanks

**Fig. 1:**
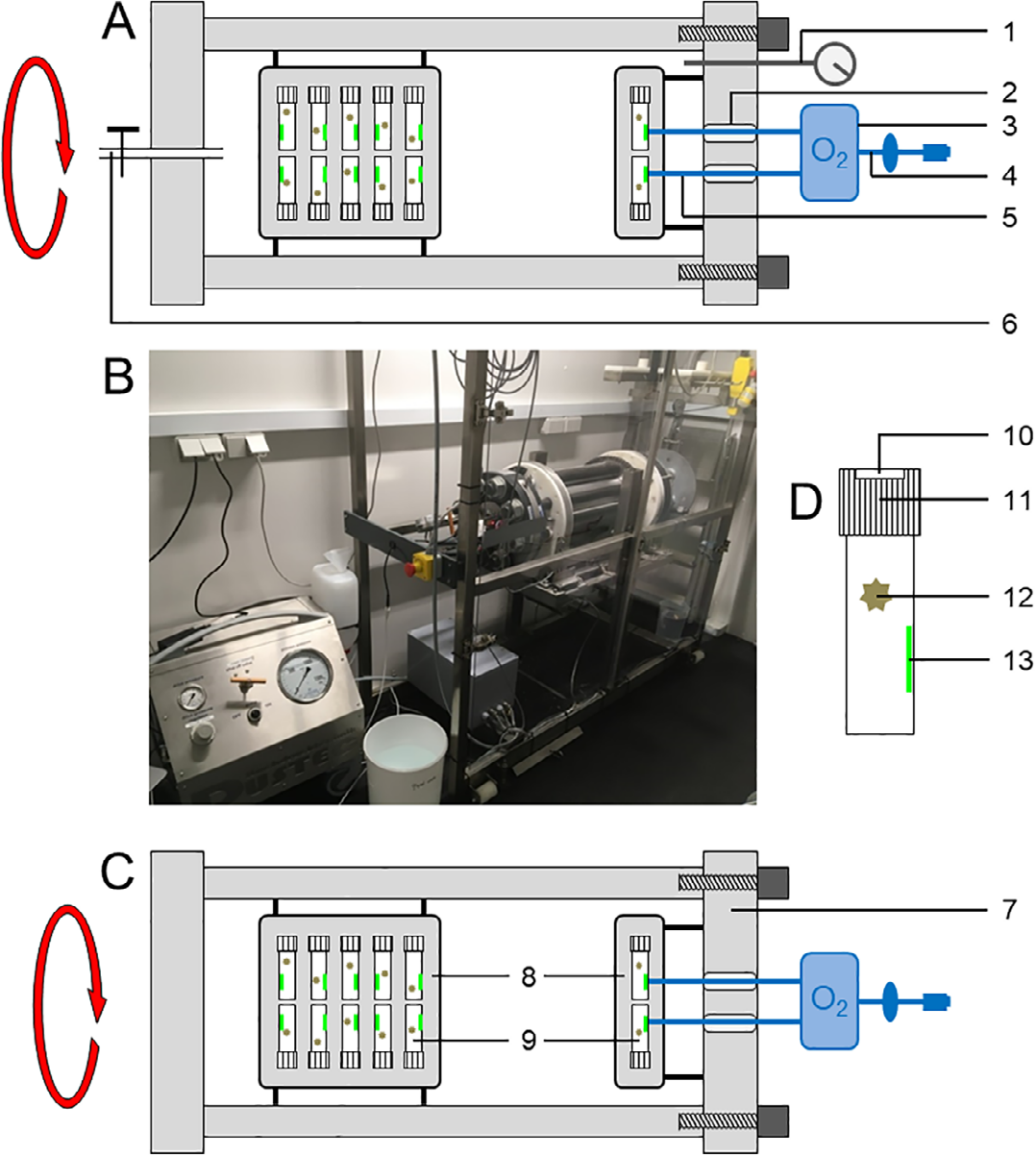
Incubation tanks and vials. (**A**) Schematic cross‐section of the pressure tank, (**B**) photograph of the pressure tank (right) and the pneumatic driven pressure pump (left), (**C**) schematic cross‐section of the control tank kept at 0.1 MPa, and (**D**) schematic of a 6‐mL incubation vial. Tanks are shown in rotating position (red arrows). Schematics illustrate the incubation principle and are not to scale. See Figs. S1 and S2 for additional pictures. 1 Pressure and temperature sensor, 2 optical feedthrough, 3 oxygen meter, 4 USB cable (with slipring) to PC, 5 fiberoptic patch cable, 6 water inlet for pressurization, 7 removable lid, 8 holding plate for incubation vials, 9 incubation vials, 10 septum, 11 screw cap, 12 diatom‐bacteria aggregate, 13 optode patch for oxygen measurements.

Pressure incubations were conducted in a cylindrical, stain‐less steel pressure tank with inner dimensions of 160 mm in diameter and 630 mm in length (Dustec GmbH, Wismar, Germany) and a maximum working pressure of 100 MPa (Figs. 1A,B,S1). The pressure tank was mounted in a custom‐made frame that allowed the tank to be tilted and locked in horizontal position. The tank could be continuously rotated around its long axis using a motor regulated by a custom‐made speed controller. The tank was pressurized with a pneumatic driven pressure pump (Dustec GmbH, Wismar, Germany).

To enable *continuous*, optical oxygen measurements inside the tank, four fiberoptic feedthroughs (M12 hermetic feedthrough, Laser Components GmbH, Olching, Germany) were mounted in the lid of the tank (Figs. 1A,S2). On the outside, the feedthroughs were connected to a four‐channel FireSting O_2_ Meter (PyroScience, Aachen, Germany) mounted on the lid, using fiberoptic patch cables. Inside the tank, the feedthroughs were connected to custom‐made, oxygen‐sensitive optode patches glued to the inside of the actual incubation vials (see below) using fiberoptic patch cables. During rotation of the pressure tank, the USB connection from the FireSting O_2_ Meter to a PC was transferred through an electrical slipring (PSC‐X12, Penlink, Hägersten, Sweden).

Control incubations at 0.1 MPa were conducted in a cylindrical tank with comparable inner dimensions, however made from transparent acrylic to allow observation of aggregates (Figs. 1C,S1). Using a benchtop roller table (Wheaton, Millville, U.S.A.), the control tank was rotated at 5 rpm as this speed would keep the aggregates continuously suspended to the extent possible inside the actual incubation vials. This rotation speed was used also for the pressure tank and for all seawater (SW), *S. marinoi* (SKEL), and aggregate (AGGR) experiments. *Continuous*, optical oxygen measurements inside the control tank were performed as described for the pressure tank.

Inside the rotating tanks, the samples were incubated in 6‐mL exetainers (Labco Ltd., Lampeter, U.K.) equipped with an internal optode patch for optical oxygen measurements (Fig. 1D). The flexible septum cap of the exetainers efficiently compensates the compression of water by up to 4% at 3°C and 100 MPa (Fine and Millero 1973). For cleaning and sterilization, exetainers equipped with an optode patch were rinsed with tap water, acid‐soaked in 1% HCl for 5 min, and rinsed again with 0.22‐*μ*m‐filtered deionized water.

### Experimental design

#### Overview of experiments

SW, SKEL, and AGGR experiments were made serially at 10 pressure levels ranging from 10 to 100 MPa. Each pressure incubation was accompanied by a control incubation at atmospheric pressure (0.1 MPa). Nonbiological controls (i.e., exetainers filled with 0.22‐*μ*m‐filtered and autoclaved seawater [RSW] prepared from Red Sea salt [Red Sea Aquatics Ltd., Cheddar, U.K.]) were included in all pressure and control incubations to be used in the data analysis of oxygen measurements (*see* Section 1.4). All experiments were carried out at 3°C and in darkness. Direct light exposure of samples containing diatoms (i.e., SKEL and AGGR) was avoided also during the preparation of the experiments.

#### Time schedule

Each experiment was divided into two consecutive phases lasting ca. 24 h each (Fig. S3): (1) the *Pressure* phase during which the effect of compression to high pressure levels was tested, and (2) the *Recovery* phase during which (a) the reversibility of potential pressure effects and/or (b) the effect of decompression were tested. Both compression and decompression of the pressure tank were achieved within 15–20 min. For each experiment, *continuous* oxygen measurements were made during the *Pressure* and *Recovery* phase, while *discrete* oxygen measurements were made at time zero (i.e., before putting the exetainers in the tank), after the *Pressure* phase and again after the *Recovery* phase (i.e., after taking the same exetainers out of the tank). A *discrete* sampling scheme was used also for microbial abundance measurements, however by sacrificing replicate exetainers at the mentioned time points. The same time schedule and sampling schemes were adopted for the control incubations while maintaining 0.1 MPa during the entire 48 h.

#### Experimental setup

For SW experiments, 200 mL nutrient‐enriched seawater was vigorously shaken for 3 × 30 s to achieve 100% air saturation and used to fill 10 exetainers with optode patches and 12 exetainers without optode patches. Exetainers with optode patches were intended for *continuous* (2 exetainers) and *discrete* oxygen measurements (3 exetainers) in both the pressure and the control tank. Exetainers without optode patches were intended for microbial abundance measurements after the *Pressure* (3 exetainers) and *Recovery* phase (3 exetainers) in both the pressure and the control tank; the leftover volume of SW was used to collect samples for time zero. For SKEL experiments, 50 mL *S. marinoi* was gently centrifuged (500 g for 5 min) and the supernatant discarded. The cell pellet was re‐suspended in 200 mL 0.22‐*μ*m‐filtered and autoclaved RSW. Aeration and filling scheme were the same as for SW samples. For AGGR experiments, aggregates were carefully transferred from the aggregate production bottle into a glass beaker filled with 250 mL RSW for washing. One aggregate was then transferred into each of 10 exetainers with optode patches and 12 exetainers without optode patches prefilled with aerated RSW, following the same scheme as described for SW and SKEL experiments.

### Respiration measurements

Before every experiment, a one‐point calibration at 100% air saturation (temperature 3°C, salinity 30) was made for all exetainers equipped with optode patches. The optode signals at 0% air saturation were occasionally checked in dithionite‐amended seawater and remained constant at values close to zero. Increasing pressure levels cause changes in temperature (minor increase), oxygen solubility (increase), and the sensitivity of the optode patches (~3% decrease per 10 MPa). The compensation of the latter effect is so far only described up to pressure levels of 60 MPa (Glud et al. 1999; Tengberg et al. 2006). Thus, an alternative procedure was implemented to correct for physical–chemical pressure effects on the *continuous* oxygen measurements. As a consequence of physical–chemical effects, the apparent (measured) oxygen concentration (O_2 app_) deviates from the theoretical (unchanged) oxygen concentration (O_2 theo_). This deviation was quantified for every time point of the *continuous* oxygen measurements in the nonbiological controls (i.e., exetainers filled with 0.22‐*μ*m‐filtered and autoclaved RSW) as F_t_ = O_2 theo_/O_2 app_. For every time point of the *continuous* oxygen measurements in the biological samples, O_2 app_ was multiplied by F_t_ to correct for the physical–chemical pressure effects and thereby arrive at O_2 corr_. Since individual optode patches may differ in the relative sensitivity response to pressure, some of the O_2 corr_ time series exhibited an additional offset (i.e., a deviation of O_2 corr_ from 100% air saturation at the start of the *Pressure* phase). This offset was manually corrected by a parallel shift of the O_2 corr_ time series for the whole *Pressure* phase, which did not change the slope of the O_2 corr_ decrease and the rate calculations.

Respiration rates were calculated from the decrease in the corrected oxygen concentrations with time. For *discrete* oxygen measurements, respiration rates were calculated for each experimental phase from the corrected start and end oxygen concentrations and the duration of the respective phase. For *continuous* oxygen measurements with linear decreases in oxygen concentration (i.e., SW experiments at 10–100 MPa, SKEL, and AGGR experiments at ≤40 MPa), respiration rates were calculated from the slope of the respective regression lines. For oxygen concentration time series that were curved at the beginning of the *Pressure* phase (i.e., SKEL and AGGR experiments at ≥50 MPa), respiration rates were calculated for the last 4 h of the *Pressure* phase. This was the longest time interval for which the decrease in oxygen concentration was linear (judging from normally distributed residuals of the linear regression analysis) across all corresponding time series. Linearity in this time interval suggested that a stable, terminal effect of pressure on respiration activity has been reached. For a few exemplary time series, shorter, near‐linear stretches in the curved part were selected for rate calculations to illustrate the initial dynamics in respiration activity in SKEL and AGGR experiments at ≥50 MPa.

### Microbial cell abundance

Microbial cell abundance was determined at *discrete* time points before and after the *Pressure* and *Recovery* phase to study pressure effects on growth (SW experiments), cell destruction (SKEL experiments), and cell detachment (AGGR experiments). SW and SKEL samples in exetainers without optode patches were thoroughly mixed before transferring aliquots of 1 mL into new sample tubes. Samples were fixed in 2% formaldehyde (final concentration) at 4°C overnight and then stored at −20°C (SW samples) or room temperature (SKEL samples). Ambient water from AGGR samples was treated the same way as SW samples except for omitting the mixing step as this would have destroyed the aggregate. At time zero of two arbitrarily selected AGGR experiments, one washed aggregate was transferred into each of three sample tubes and stored at −20°C. In SW samples and in ambient water from AGGR samples, microbial abundance was quantified by flow cytometry; in SKEL samples, diatom abundance was quantified by microscopy; in aggregates, bacterial and diatom abundance was estimated by PLFA analysis following the procedure described in Elvert et al. (2003) but with modifications. In brief, centrifuged samples were extracted using the modified Bligh and Dyer protocol by Sturt et al. (2004), giving a total lipid extract from which FA moieties in PLFAs were released by saponification and converted to fatty acid methyl esters (FAMEs) using BF_3_ in methanol. FAME abundances, determined via the internal FA standard 2Me‐C_18:0_ added prior to extraction, and identities were investigated by gas chromatography (GC) coupled to flame ionization detection (ThermoFinnigan) and by GC–mass spectrometry (GC–MS, ThermoFinnigan Trace GC coupled to Trace MS), respectively, using temperature settings and chromatographic columns described in Aepfler et al. (2019).

For flow cytometric analysis, the fixed samples were stained with SYBR® Gold Nucleic Acid Gel Stain (Thermo Fisher Scientific, Waltham, U.S.A.) for 30 min and run on a BD FACSAria® III (Biosciences, San Jose, U.S.A.). Flow cytometric data were analyzed using the Flowing Software by Perttu Terho (University of Turku, Finland). Dot plots of fluorescence vs. side scatter were used to define gates for specific and unspecific signals belonging to stained microbial cells and unidentified particles, respectively. The dot plots were further evaluated for the presence of distinct cell populations that could be assigned to Bacteria and Archaea or diatoms.

For microscopic counts, the fixed SKEL samples were thoroughly homogenized and transferred to a Fuchs‐Rosenthal counting chamber. For each SKEL sample, 10 grids corresponding to a volume of 0.2 *μ*L each were counted on a Zeiss Axio Lab.A1 light microscope at 100× magnification.

### Aggregate characteristics

The volume of each aggregate used for experimentation was approximated from its projected circular area and equivalent radius from which the volume of a sphere was calculated. The projected area was obtained from an image taken while the aggregate was sinking along the vertical axis of a 6‐mL exetainer using the image‐processing program ImageJ (W.S. Rasband, NIH, Bethesda). The elemental composition of 20 randomly selected aggregates of known volume was analyzed on a Na 1500 elemental analyzer (Fisons, Loughborough, U.K.). The sinking speed of 16 randomly selected aggregates of known volume was determined in a glass cylinder (inner diameter: 7.5 cm) filled with stagnant and temperature‐equilibrated (3°C) seawater (salinity 30) by taking the time for each aggregate to sink through the water column (height: 25 cm) (Stief et al. 2016).

## Results

### Pressure effect on respiration revealed by *discrete* oxygen measurements

**Fig. 2:**
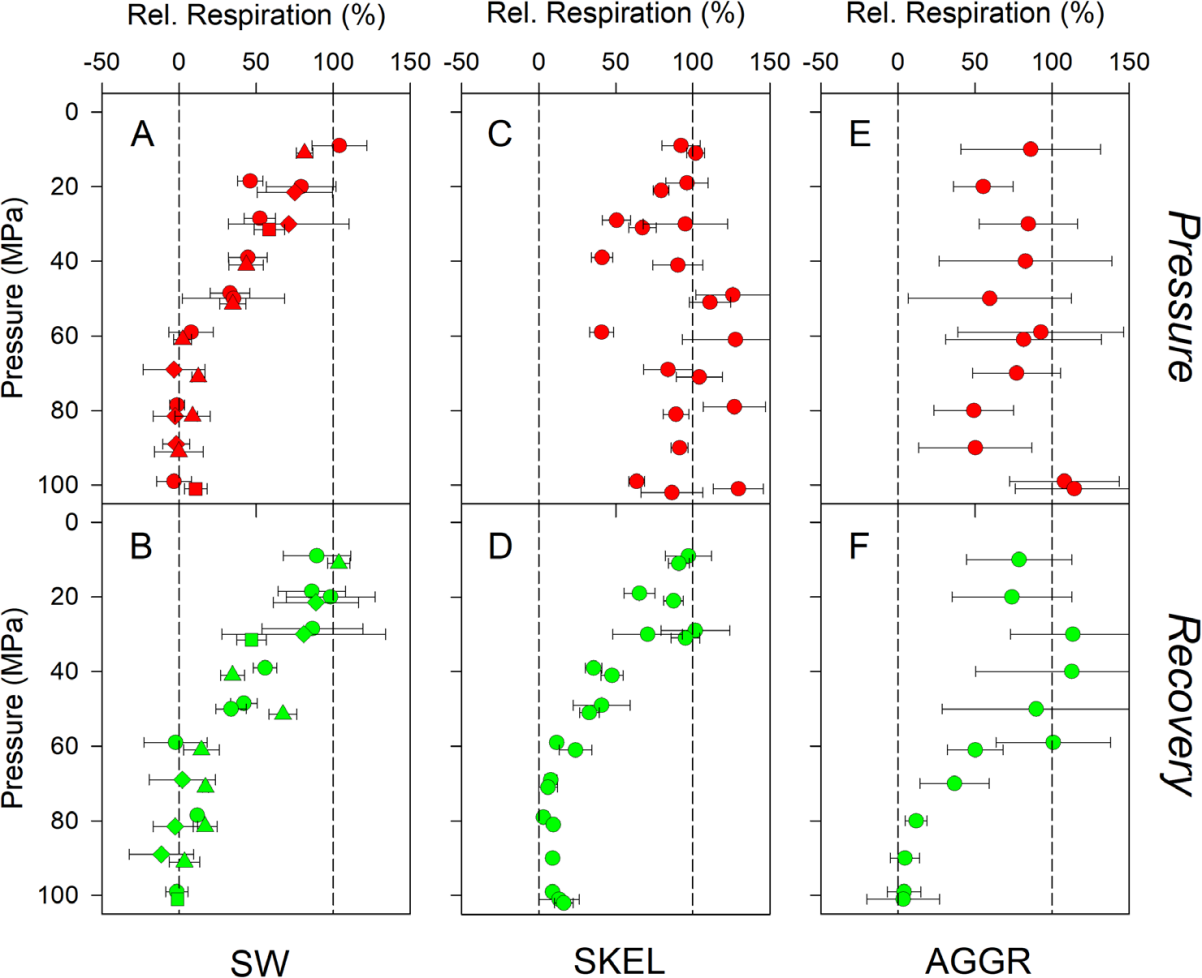
Pressure effect on respiration revealed by *discrete* oxygen measurements. Results for nutrient‐enriched seawater (SW), axenic *S. marinoi* cultures (SKEL), and diatom‐bacteria aggregates (AGGR) during the (**A**,**C**,**E**) *Pressure* phase and (**B**,**D**,**F**) *Recovery* phase are shown. (**A**,**B**) four independently produced SW batches are represented by different symbols. Relative respiration rates were calculated as the ratio of respiration rates at high over atmospheric pressure expressed in %. Values of 0% and 100% (dashed vertical lines) correspond to complete and no inhibition of respiration at high pressure, respectively. For aggregates, respiration rates were normalized to aggregate volume before calculating relative respiration rates. Means ± SD of 3 (SW and SKEL) and 6 (AGGR) replicates are shown. For multiple experiments at the same pressure level, data points were slightly offset for better visibility.

In control incubations of the seawater (SW) experiments, respiration rates were 0.74 ± 0.50 *μ*M O_2_/h (mean ± SD, *n* = 24 experiments). Relative respiration rates calculated as the ratio of respiration rates at high over atmospheric pressure gradually decreased from ~100% at 10 MPa (i.e., no inhibition by pressure) to ~0% at 60 MPa (i.e., complete inhibition by pressure) during the *Pressure* phase (Fig. 2A). Pressure levels of 60–100 MPa completely inhibited respiration. This general pattern was observed for the four different batches of nutrient‐enriched seawater used for incubations. Additionally, this general pattern remained the same during the *Recovery* phase (Fig. 2B). However, a number of incubations resulted in higher relative respiration rates than observed during the *Pressure* phase, indicating that the pressure effect was reversible to some extent.

In control incubations of the *S. marinoi* (SKEL) experiments, respiration rates were 0.82 ± 0.38 *μ*M O_2_/h (mean ± SD, *n* = 22 experiments). Relative respiration rates varied between ~40% and ~130% during the *Pressure* phase, with no noticeable dependence on the absolute pressure level (Fig. 2C). Thus, the *discrete* oxygen measurements suggest that dark respiration by *S. marinoi* is (1) not completely inhibited by high pressure levels and (2) in some cases even stimulated by high pressure levels (but see section on *continuous* oxygen measurements). The pressure dependence of relative respiration rates during the *Recovery* phase was very similar to that observed in SW samples, with partial and complete inhibition following pressure incubations at 10–50 and 60–100 MPa, respectively (Fig. 2D).

In control incubations of the aggregate (AGGR) experiments, total respiration rates in the exetainer were 1.06 ± 0.75 *μ*M O_2_/h (mean ± SD, *n* = 92 aggregate incubations). Since by far the most diatom and bacterial cells were present in the aggregate and not in the ambient water (see *Pressure effect on microbial cell abundance*), respiration activity was allocated to the aggregate alone. The above total respiration rates therefore amounted to 0.15 ± 0.11 *μ*mol O_2_/aggregate × d or 6.0 ± 3.1 nmol O_2_/mm^3^ × d. Due to size differences between aggregates used for pressure and control incubations, the relative respiration rates were calculated from the aggregate‐volume‐specific respiration rates. Relative respiration rates varied between ~40% and ~90% at 10–90 MPa during the *Pressure* phase, indicating partial inhibition (Fig. 2E). In two incubations at 100 MPa, the *discrete* oxygen measurements suggested that aggregate respiration was slightly stimulated relative to incubations at atmospheric pressure (but see the following on *continuous* oxygen measurements). During the *Recovery* phase, no inhibition was observed following pressure incubations at 30–60 MPa, partial inhibition was observed following pressure incubations at 10–20 and 60–70 MPa, and complete inhibition was observed following pressure incubations at 80–100 MPa (Fig. 2F).

### Pressure effect on respiration revealed by *continuous* oxygen measurements

#### Shape of oxygen time series

**Fig. 3:**
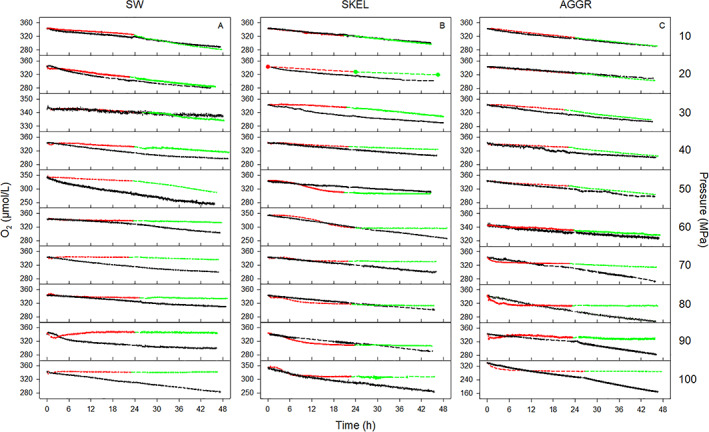
Pressure effect on respiration revealed by *continuous* oxygen measurements. Oxygen concentration time series for (**A**) nutrient‐enriched seawater (SW), (**B**) axenic *S. marinoi* cultures (SKEL), and (**C**) diatom‐bacteria aggregates (AGGR) in rotating pressure (red and green line) and control tanks (black line) are shown. Oxygen concentration was logged every 1 min for ca. 24 h each during the *Pressure* (red line) and *Recovery* phase (green line) using optical oxygen measurements. Note that incubations in the control tank were run at atmospheric pressure during both the *Pressure* and the *Recovery* phase. Dashed lines bridge interruptions of data logging between *Pressure* and *Recovery* phase and due to logging failure.

Oxygen concentration decreased linearly with time in almost all SW, SKEL, and AGGR control incubations at 0.1 MPa (Fig. 3). The slopes of the decrease in oxygen concentration were similar for the two phases of the respective pressure experiment, indicating no major change in respiration activity with time. Oxygen concentration decreased linearly with time also in most SW pressure incubations at 10–100 MPa and in SKEL and AGGR pressure incubations at 10–50 MPa (Fig. 3A–C).

In contrast, curved (i.e., nonlinear) oxygen time series were observed in SKEL and AGGR pressure incubations at 50–100 and 60–100 MPa, respectively (Fig. 3B,C). During the *Pressure* phase, these curved oxygen time series displayed two typical sequences of fast and slow decreases in oxygen concentration: (1) very slow–fast–very slow (SKEL) or (2) very fast–very slow (AGGR) (Fig. S4). Rate calculations for the curved part of these oxygen time series revealed up to 15‐fold changes in respiration rate over short time intervals (Fig. S4B,C). During the *Recovery* phase of the same SKEL and AGGR experiments, the oxygen time series were linear and the slopes corresponded to extremely low respiration rates.

Overlaying *continuous* with *discrete* oxygen measurements generally revealed a good match between the two approaches (Fig. S4). While the nonlinear features of oxygen consumption were only captured by the *continuous*, but not the *discrete* oxygen measurements, the cumulative oxygen consumption during the *Pressure* phase as quantified by the two approaches was not significantly different with few exceptions (Table S3).

#### Relative respiration rates

**Fig. 4:**
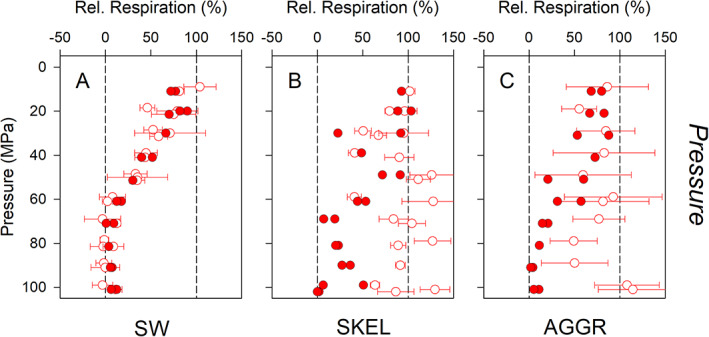
Comparison of *discrete* and *continuous* oxygen measurements. Relative respiration rates for nutrient‐enriched seawater (SW), axenic *S. marinoi* cultures (SKEL), and diatom‐bacteria aggregates (AGGR) during the *Pressure* phase are shown. Relative respiration rates based on *continuous* oxygen measurements (closed symbols) were calculated for the last 4 h of the *Pressure* phase; relative respiration rates based on *discrete* oxygen measurements (open symbols) are the same as those presented in Fig. 2. Values of 0% and 100% (dashed vertical lines) correspond to complete and no inhibition of respiration at high pressure, respectively. Means ± SD of 3–6 replicates (*discrete* oxygen measurements) or single values (*continuous* oxygen measurements) are shown. See results section and Table S4 for statistics. For multiple experiments at the same pressure level, data points were slightly offset for better visibility.

For the last 4 h of the *Pressure* phase, the decrease in oxygen concentration was linear with time for all sample types, suggesting a stable, terminal effect of pressure on respiration activity has been reached. Relative respiration rates were hence calculated as the ratio of slopes of oxygen decrease at high over atmospheric pressure and compared with rates obtained from *discrete* oxygen measurements (Fig. 4). For SW pressure incubations, the rates obtained from *continuous* oxygen measurements were not significantly different from the rates obtained from *discrete* oxygen measurements across the full range of pressure levels tested (Fig. 4A; Table S4). For SKEL pressure incubations, this match was only observed at pressure levels of 10–60 MPa, while significantly lower rates were consistently obtained for high pressure levels of 70–100 MPa using the *continuous* oxygen time series (Fig. 4B; Table S4). Similarly, for AGGR pressure incubations, significantly lower rates were consistently obtained for high pressure levels of 70–100 MPa (Fig. 4C; Table S4). The latter two observations indicate that toward the end of the *Pressure* phase, respiration in SKEL and AGGR samples was more strongly inhibited than could be read from the data obtained through *discrete* oxygen measurements. Importantly, in all high‐pressure incubations, respiration was completely inhibited toward the end of the *Pressure* phase at 50–100 MPa (SW and SKEL samples) and 70–100 MPa (AGGR samples), and remained completely inhibited also during the *Recovery* phase.

### Pressure effect on microbial cell abundance

**Fig. 5:**
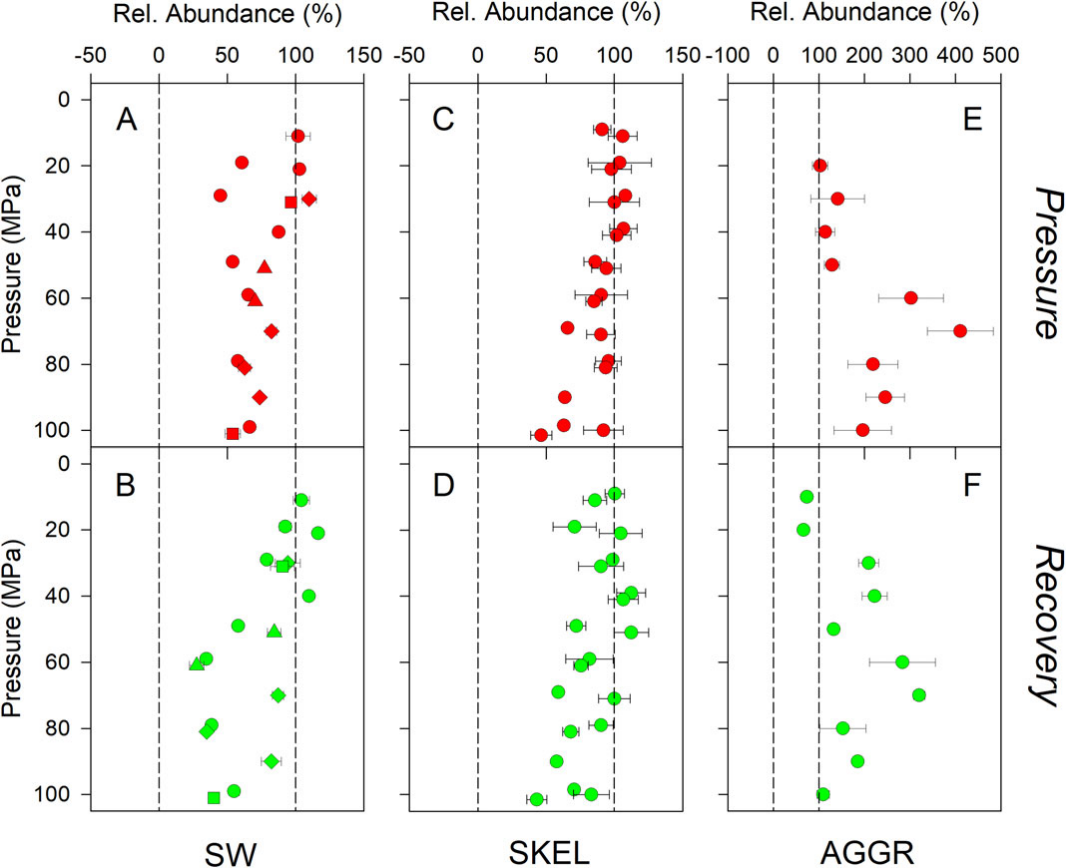
Pressure effect on microbial cell abundance. Results for nutrient‐enriched seawater (SW), axenic *S. marinoi* cultures (SKEL), and diatom‐bacteria aggregates (AGGR) at the end of the (**A**,**C**,**E**) *Pressure* phase and (**B**,**D**,**F**) *Recovery* phase are shown. For SW and SKEL experiments, bacteria and diatoms were enumerated, respectively; for AGGR experiments, free‐living bacteria in the ambient water were enumerated. Relative abundance was calculated as the ratio of cell abundance at high over atmospheric pressure expressed in %. Values of higher and lower than 100% (dashed vertical line) correspond to a relative increase and decrease in cell abundance at high pressure, respectively. Means ± SD of 3 replicates are shown. For multiple experiments at the same pressure level, data points were slightly offset for better visibility.

For SW experiments, the flow‐cytometry data revealed that the microbial community of the seawater was dominated by Bacteria and/or Archaea (henceforth referred to as ‘bacteria’) while the abundance of diatoms and other microalgae was lower than the detection limit of 10^5^ cells/L. The bacterial abundance at time zero of the SW experiments was 6.5 × 10^8^ ± 4.6 × 10^8^ cells/L (mean ± SD, *n* = 17 experiments). The relative bacterial abundance varied between ~50% and ~100% at the end of the *Pressure* phase and tended to be lower at higher pressure levels (Fig. 5A). This general pattern remained the same at the end of the *Recovery* phase (Fig. 5B). Relative abundances <100% result from inhibited growth and/or from promoted cell destruction at high vs. low pressure levels. For those SW experiments in which significant net growth was observed in the control incubations at 0.1 MPa (one‐sample *t*‐test, *p* < 0.05), growth was strongly inhibited at pressure levels of 20–100 MPa during the *Pressure* phase (Fig. S5A). During the *Recovery* phase, growth was strongly promoted following pressure incubations at 10–40 MPa, but remained inhibited following pressure incubations at 50–100 MPa (Fig. S5B). Occasionally, decreases in absolute cell abundance were observed at high pressure levels, which supported that bacterial cell destruction was also promoted by pressure.

In SKEL experiments, the diatom abundance at time zero was 2.5 × 10^8^ ± 2.5 × 10^8^ cells/L (mean ± SD, *n* = 20 experiments). The relative diatom abundance varied between ~50% and ~100% at the end of the *Pressure* phase, with values lower than 100% occurring at pressure levels of 50–100 MPa only (Fig. 5C). This general pattern remained the same at the end of the *Recovery* phase (Fig. 5D). Net growth of *S. marinoi* in the control incubations at 0.1 MPa was not significantly different from zero in any of the experiments (one‐sample *t*‐test, *p* > 0.05). Decreases in absolute cell abundance were rarely observed at high pressure levels, but diatoms which had apparently lost their cells contents (i.e., empty frustules) were regularly noted under the microscope and not included in the cell counts.

For the ambient water of AGGR experiments, the flow‐cytometry data revealed that the abundance of diatoms was lower than the detection limit of 10^5^ cells/L and that bacteria dominated the free‐living microbial community. Bacterial abundance reached 1.2 × 10^8^ ± 0.9 × 10^8^ cells/L (mean ± SD, *n* = 10 experiments) after 24 h of incubation at 0.1 MPa (note that samples could not be collected at time zero). Relative bacterial abundance varied between ~100% and ~400% at the end of the *Pressure* phase and was particularly high at pressure levels of 60–100 MPa (Fig. 5E). This general pattern remained the same at the end of the *Recovery* phase (Fig. 5F). Relative bacterial abundances >100% result from more extensive cell detachment from the aggregate at high vs. low pressure levels, since aggregates were incubated in initially bacterial‐cell‐free seawater.

Diatom and bacterial abundance in six average‐sized aggregates (~24 mm^3^) was estimated by combining flow‐cytometric bacterial counts, microscopic diatom counts, and PLFA data (Table S2). The *S. marinoi* culture used for aggregate production contained 1.3 × 10^8^ diatom cells/L and 1319 *μ*g C_20:5_/L, corresponding to 9920 fg C_20:5_/diatom cell. The natural seawater used for aggregate production contained 9.1 × 10^7^ bacterial cells/L and 49 ng iC_15:0_/L, corresponding to 0.53 fg iC_15:0_/bacterial cell. Thus, the PLFA contents of C_20:5_ and iC_15:0_ in the aggregates of 505 ± 397 ng and 38 ± 25 ng, respectively, corresponded to 5.1 × 10^4^ ± 4.0 × 10^4^ diatom cells/aggregate and 7.1 × 10^7^ ± 4.7 × 10^7^ bacterial cells/aggregate (mean ± SD, *n* = 6 aggregates).

Across all AGGR experiments and sampling time points during incubations at 0.1 MPa, bacterial abundance in the ambient water was 1.4 × 10^8^ ± 1.3 × 10^8^ cells/L, which corresponded to 1.2% ± 1.1% of total bacteria in the aggregates (mean ± SD, *n* = 20 measurements). Exposure to pressure levels of 60–100 MPa increased bacterial abundance in the ambient water by 1.2–2.0 × 10^8^ cells/L, while the diatom abundance in the ambient water remained below detection. Hence, the additional detachment of bacterial cells upon pressure exposure amounted to 1.0%–1.6% of total bacteria in the aggregates.

## Discussion

### *Continuous* vs. *discrete* oxygen measurements

In most experiments and for all sample types, linear or close‐to‐linear decreases in oxygen concentration were measured during the *Pressure* and *Recovery* phase. Linear oxygen time series translate into constant respiration rates which are, within the error margins, identical for *continuous* and *discrete* oxygen measurements. In contrast, the curved oxygen time series observed for SKEL and AGGR samples exposed to pressure levels of 50–100 MPa correspond to a complex sequence of high and low respiration activity. Presumably, such high pressure levels differentially stimulate or inhibit unidentified metabolic processes in the diatom *S. marinoi* (since curved oxygen time series were not observed in the diatom‐free SW samples), which is accompanied by increased and decreased oxygen consumption, respectively. This sequence of high and low respiration activity ended in (almost) complete inhibition of respiration activity in the second half of the *Pressure* phase, which was not captured by the *discrete* oxygen measurements that falsely implied respiration activity throughout the entire *Pressure* phase. Notably, the *continuous* oxygen measurements provide information on microbial activities in real time and not retrospectively like the *discrete* oxygen measurements, which allows immediate interpretation and interactive experimentation.

### Pressure effects on activity and abundance of surface‐water microbial communities

Common to all surface‐water microorganisms tested were (1) weak to moderate pressure effects at 10–40 MPa as opposed to strong or fatal pressure effects at 50–100 MPa, (2) stronger pressure effects on respiration than on abundance, and (3) occasional recovery of respiration activity following pressure incubation at 10–40 MPa. Taken together, pressure acts primarily on the metabolism of surface‐water microorganisms and to a lesser extent on their abundance. This applies also to *S. marinoi*, which is the first aggregate‐forming diatom pressure‐tested alive, while previous studies used microalgal detritus as organic substrate for prokaryotes (Tamburini et al. 2006; Riou et al. 2018). The dark respiration activity by *S. marinoi* is likely fueled by the aerobic degradation of organic storage compounds. Within diatom aggregates, live *S. marinoi* thus contribute actively to organic carbon degradation, while dead *S. marinoi* are degraded by bacteria.

The weak to moderate pressure effects at 10–40 MPa went hand in hand with the occasional recovery of respiration activity, which indicates that at least a subset of the microorganisms and microbial communities survived the pressure exposure to 10–40 MPa. In contrast, the strong pressure effects at 50–100 MPa precluded the recovery of respiration activity by the microbial communities and diatom species investigated here.

The observed lower relative abundances of bacteria (SW experiments) and diatoms (SKEL experiments) at high pressure levels might be due to impeded growth and cell destruction, respectively. Irrespective of pressure effects, bacteria may readily grow under the dark and cold experimental conditions as long as substrates and nutrients are available and grazers do not suppress them efficiently. Bacterial growth in the control incubations at 0.1 MPa was indeed observed in 14 out of 17 SW experiments and was impeded at pressure levels >20 MPa. In contrast, for the phototrophic diatoms, the dark and cold experimental conditions represent a substantial stress due to the absence of light. Dark respiration by *S. marinoi* did not allow net growth in any of the control incubations and is moreover expected to cease once organic storage compounds are used up. Under natural conditions in the ocean, the diatom cells have by then either transitioned into a resting stage or they are bound to die. Instead of impeding the growth of dark‐incubated diatoms, high pressure levels may rather destroy diatom cells mechanically, which is supported by (1) the occurrence of empty frustules and the decrease in absolute diatom abundance and (2) the increase in ambient DOC concentrations in pressure incubations (data not shown).

Differential pressure responses of surface‐water microorganisms included (1) gradually decreasing relative respiration rates in SW samples and to some extent in AGGR samples, but not in SKEL samples (at 10–50 MPa), (2) curved oxygen time series in SKEL and AGGR samples, but not in SW samples (at 50–100 MPa), and (3) occasional relative stimulation of respiration activity in SKEL and AGGR samples, but not in SW samples (at 10–100 MPa). Differential pressure responses were thus obviously related to the presence or absence of the diatom *S. marinoi*.

A gradual, pressure‐dependent decrease in metabolic activities such as nucleic acid and protein synthesis, aminopeptidase activity, and respiration has previously been observed for surface‐water microbial communities (Turley 1993; Tholosan et al. 1999; Tamburini et al. 2006; de Jesus Mendes et al. 2007). The negative correlation between pressure level and metabolic activity may be explained by a growing number of community members that are inhibited by increasing pressure levels. The SW and AGGR samples investigated here each host an uncharacterized prokaryotic diversity that forms the basis for such a gradual pressure response. In contrast, the SKEL samples represent a mono‐specific microbial population for which a relatively fixed, inhibitory pressure level may be expected. However, the response by *S. marinoi* to increasing pressure levels was rather erratic, maybe owing to different physiological states of the cultures between experiments.

Curved oxygen time series in pressure incubations have previously been observed and were either ascribed to temperature effects (Boyd et al. 2015) or remained uncommented (Garel et al. 2019). Here, the occurrence of curved oxygen time series was limited to *S. marinoi*‐containing samples. In this diatom species, very high pressure levels seem to elicit a complex metabolic response that comprises periods of high and low oxygen consumption. Bacterial respiration, potentially stimulated by DOC leaking from pressure‐exposed diatom cells, can be ruled out as a driver of the observed oxygen dynamics because the *S. marinoi* cultures were axenic. Additionally, temperature varied by <1°C and its effect on oxygen measurements was anyway corrected for via the nonbiological controls. This leaves high pressure levels as the most likely trigger of a complex metabolic response by *S. marinoi*, which eventually resulted in complete inhibition of its respiration activity. It remains to be explored, if the ceased respiration activity means that *S. marinoi* has entered a resting stage or eventually died. The pressure response by diatom‐containing aggregates was different from that by axenic *S. marinoi* cultures, in that the initial phase of low respiration activity was missing. Here, heterotrophic bacteria within the aggregates might have been able to make use of the DOC released from lysing diatom cells (Tamburini et al. 2006), which masked the lower respiration activity by *S. marinoi*. However, this is difficult to reconcile with the immediate inhibition of respiration activity in SW samples exposed to pressure levels of >50 MPa. Respiration activity in the diatom‐containing SKEL and AGGR samples was occasionally stimulated upon pressure exposure. It may be speculated that high pressure levels trigger the aerobic degradation of organic storage compounds, such as laminarin and lipids, to initiate the transition of vegetative cells into resting cells or spores.

In aggregate incubations, a pressure‐induced increase in relative abundance of bacteria in the ambient water was observed. Since the aggregates had been incubated in filtered and autoclaved seawater, the free‐living bacteria must have been detached from the aggregates, and this to a larger extent at high pressure levels. Cell detachment corresponds to a loss term for pressure‐exposed, sinking aggregates, but did not exceed 1.6% of total bacteria in the model aggregates studied here. It needs to be explored, if the slower pressure increase that natural aggregates experience during their descent is accompanied by cell detachment to a similar extent.

### Organic carbon preservation in pressure‐exposed model aggregates

The diatom‐bacteria aggregates studied here were relatively compact and thus fast‐sinking compared to natural phytodetritus aggregates (Alldredge and Gotschalk 1989). The compactness may originate from occasional collisions of the aggregates with the walls of the aggregate production bottles and exetainers during production, experimental handling, and incubation (Jackson 2015). A continuous solid body rotation prohibiting these collisions was unlikely to establish because of the shape of bottles and exetainers and the direction of rotation (Shanks and Edmondson 1989; Ploug et al. 2010). However, during the incubations, the aggregates were suspended essentially always, allowing for diffusive oxygen exchange across the entire aggregate surface. Hence, the total and carbon‐specific respiration rates of an average‐sized model aggregate (~24 mm^3^) were very similar to those reported for other laboratory‐produced diatom aggregates (Iversen and Ploug 2013).

The pressure‐dependent inhibition of respiration activity observed for the model aggregates studied here permits only limited conclusions on the extent of organic carbon preservation within natural aggregates exposed to high pressure levels. This is mainly because the pressure history of the model aggregates does not properly reflect the likely pressure history of natural aggregates. First, in each experiment, only one fixed pressure level was tested rather than a gradual increase in pressure level. Second, even the highest pressure levels tested were reached within 15–20 min rather than after several days of sinking. As a consequence, the experimental incubations did not allow for an acclimatization of the pressure‐exposed microorganisms to a relatively slow increase in pressure. In the absence of such microbial acclimatization, the respiration activity associated with the model aggregates was reduced by 50%–100% when pressure levels of 50–100 MPa were applied. This suggests that organic matter degradation within sinking aggregates can be substantially reduced at water depths of 5 km and deeper, allowing more nondegraded organic carbon to reach abyssal and hadal depths than without pressure effects. This needs to be confirmed by experiments in which the pressure level is gradually increased over realistic time scales. For an average‐sized model aggregate with a sinking velocity of 495 m/d (Fig. S6A), the simulated descent to 10 km water depth would take ~20 d and involve a pressure increase of 5 MPa/d. Based on the average respiration rate measured in control incubations at 0.1 MPa, only ~30% of the initial organic carbon content of 4.3 *μ*mol C (Fig. S6B) would arrive at 10 km depth, but likely more, if pressure reduces also the microbial degradation of naturally sinking aggregates.

Natural diatom aggregates experience even more changes during their descent, such as (1) fragmentation, (2) grazing, (3) de novo colonization by free‐living microorganisms at greater water depth, and (4) chemosynthetic production of new biomass (Simon et al. 2002; Aristegui et al. 2009; Thiele et al. 2015; Turner 2015). For sinking aggregates, a dynamic exchange between free‐living and aggregate‐associated microbial communities is predicted (Kiørboe et al. 2002). However, the de novo colonization by free‐living microorganisms appears most intense in surface waters (Thiele et al. 2015) and rather limited in deep waters (Eloe et al. 2011; Mestre et al. 2018). The pressure tolerance of newly attached microorganisms will likely increase with depth (Cario et al. 2019), which suggests weaker or no inhibition of aggregate degradation by pressure. However, also the newly attached microorganisms will encounter higher pressure levels to which they are not adapted (Tholosan et al. 1999; Grossart and Gust 2009; Marietou and Bartlett 2014), especially if the sinking speed of mineral‐ballasted aggregates increases in the bathypelagic zone (Berelson 2002; Fischer and Karakas 2009). Irrespective of the ultimate extent of inhibition of organic carbon degradation in sinking aggregates, the pressure‐adapted microbial communities in hadal sediments possess the proven potential to degrade the settling aggregates at high rate (Glud et al. 2013; Glud et al. 2021).

## Conflict of interest

None declared.

## Supporting information

**Appendix S1:** Supporting Information
