# Supplementary material for: Respiration by “marine snow” at high hydrostatic pressure: Insights from continuous oxygen measurements in a rotating pressure tank
Source: Limnol Oceanogr. 2021 May 14;66(7):2797–809. doi: 10.1002/lno.11791 (PMC8359982; doi:10.1002/lno.11791)
Supplement: Supplementary file 1 — Appendix S1: Supporting Information [file LNO-66-2797-s001.docx]

Supplementary Information

Respiration by ‘marine snow’ at high hydrostatic pressure:

Insights from continuous oxygen measurements in a rotating pressure tank

Peter Stief^1*^, Marcus Elvert^2^, and Ronnie N. Glud^1,3,4^

# ^1^HADAL & Nordcee, Department of Biology, University of Southern Denmark, Odense, Denmark

^2^MARUM - Center for Marine Environmental Sciences and Faculty of Geosciences,

University of Bremen, Bremen, Germany

^3^Danish Institute for Advanced Study (DIAS), University of Southern Denmark, Fioniavej 34, 5230 Odense, Denmark

^4^Department of Ocean and Environmental Sciences, Tokyo University of Marine Science and Technology, Tokyo, Japan

*Correspondence:

# University of Southern Denmark, Department of Biology, HADAL & Nordcee, Campusvej 55, 5230 Odense M, Denmark; Phone: +45 6550 7980, E-mail: peterstief@biology.sdu.dk

**Table S1: Preparation of nutrient-enriched seawater.** The concentrations (in µmol/L) measured in L1-amended seawater were used to calculate the amount of NH_4_^+^ and PO_4_^3-^ needed to achieve an elemental composition of C:N:P = 106:16:1 in the nutrient-enriched seawater.

| **Batch No.** | **L1-amended seawater**  **(actual value)** | | | | **Nutrient-enriched seawater**  **(desired value)** | | | |
| --- | --- | --- | --- | --- | --- | --- | --- | --- |
|  | **DOC** | **NO_3_^-^** | **NH_4_^+^** | **PO_4_^3-^** | **DOC** | **NO_3_^-^** | **NH_4_^+^** | **PO_4_^3-^** |
| 1 | 248 | 18 | 0.2 | <0.1 | 248 | 18 | 19 | 2.5 |
| 2 | 253 | 24 | 0.3 | <0.1 | 253 | 24 | 14 | 2.5 |
| 3 | 311 | 20 | 0.1 | <0.1 | 311 | 20 | 27 | 3.0 |
| 4 | 281 | 11 | 0.2 | <0.1 | 281 | 11 | 31 | 3.0 |

L1 = spent growth media of *S. marinoi* cultures, DOC = dissolved organic carbon

**Table S2: PLFA raw data.** Fatty acid concentrations, in order of GC retention times, in the seawater and the *S. marinoi* culture used to produce the diatom-bacteria aggregates A1-A6. The specificity of FAs for bacteria or algae or both is given. The diatom-specific C_20:5_ and the bacteria-specific iC_15:0_ were used for estimating diatom and bacterial cell numbers in the aggregates, respectively (grey-shaded rows). The bacteria-specific iC_15:0_ was used to assess axenia of the *S. marinoi* culture. It should be noted that its signal was indistinguishable from laboratory blank runs.

| **Fatty acid** | **Bacteria/ Algae** | **Seawater**  **(ng/L)** | ***S. marinoi***  **(µg/L)** | **A1** | **A2** | **A3** | **A4** | **A5** | **A6** |
| --- | --- | --- | --- | --- | --- | --- | --- | --- | --- |
|  |  |  |  | **(ng/aggregate)** | | | | | |
| iC_14:0_ | Bacteria | 23 | 0.16 | 14 | 22 | 33 | 9 | 12 | 9 |
| C_14:0_ | Bact./Algae | 204 | 840 | 148 | 682 | 661 | 821 | 517 | 70 |
| iC_15:0_ | Bacteria | 49 | 0.72 | 22 | 13 | 15 | 47 | 62 | 70 |
| aiC_15:0_ | Bacteria | 56 | 1.61 | 25 | 35 | 29 | 44 | 45 | 33 |
| C_15:0_ | Bact./Algae | 50 | 23 | 22 | 33 | 43 | 38 | 60 | 26 |
| iC_16:0_ | Bacteria | 7 | 0.00 | 8 | 33 | 32 | 41 | 26 | 6 |
| C_16:4_ | Algae | 0 | 114 | 23 | 100 | 96 | 123 | 77 | 19 |
| C_16:3_ | Algae | 0 | 358 | 167 | 1055 | 1197 | 604 | 331 | 80 |
| C_16:2_ | Algae | 0 | 1016 | 136 | 726 | 712 | 639 | 398 | 169 |
| C_16:1 (n−9)_ | Bact./Algae | 18 | 215 | 39 | 131 | 44 | 126 | 123 | 57 |
| C_16:1 (n−7)_ | Bact./Algae | 554 | 985 | 192 | 743 | 678 | 659 | 442 | 178 |
| C_16:0_ | Bact./Algae | 1987 | 907 | 1674 | 1922 | 1849 | 2476 | 2081 | 1706 |
| iC_17:0_ | Bacteria | 5 | 0.22 | 22 | 16 | 16 | 4 | 6 | 5 |
| aiC_17:0_ | Bacteria | 9 | 0.42 | 12 | 8 | 9 | 8 | 13 | 8 |
| C_17:0_ | Bact./Algae | 19 | 5 | 0 | 0 | 32 | 27 | 26 | 0 |
| C_18:3_ | Algae | 0 | 20 | 24 | 148 | 150 | 55 | 36 | 0 |
| C_18:4_ | Algae | 29 | 84 | 26 | 169 | 144 | 129 | 109 | 17 |
| C_18:2_ | Algae | 67 | 42 | 45 | 185 | 200 | 257 | 188 | 65 |
| C_18:1 (n−9)_ | Bact./Algae | 956 | 92 | 9 | 274 | 307 | 479 | 502 | 379 |
| C_18:1 (n−7)_ | Bact./Algae | 117 | 14 | 26 | 96 | 79 | 58 | 48 | 55 |
| C_18:0_ | Bact./Algae | 1546 | 164 | 1884 | 1334 | 1303 | 1691 | 1630 | 1563 |
| C_20:4_ | Algae | 0 | 8 | 5 | 16 | 25 | 25 | 25 | 0 |
| C_20:5_ | Algae | 0 | 1319 | 134 | 876 | 1017 | 553 | 436 | 12 |
| C_20:0_ | Bact./Algae | 15 | 3 | 25 | 21 | 20 | 26 | 24 | 22 |
| C_22:6_ | Algae | 0 | 70 | 0 | 27 | 62 | 32 | 34 | 0 |

**Table S3: Statistical comparison of cumulative oxygen consumption during the *Pressure* phase** **of SW, SKEL, and AGGR experiments calculated from *discrete* versus *continuous* oxygen measurements.**

| **SW Experiment** | **Pressure (MPa)** | ***p*** |  | **SKEL Experiment** | **Pressure (MPa)** | ***p*** |  | **AGGR Experiment** | **Pressure (MPa)** | ***p*** |
| --- | --- | --- | --- | --- | --- | --- | --- | --- | --- | --- |
| SW V 100  SW V 100  SW III 200c  SW III 200c  SW III 300c  SW III 300c  SW V 400  SW V 400  SW V 500c  SW V 500c  SW V 600  SW V 600  SW V 400  SW V 700  SW V 800  SW V 800  SW V 900  SW V 900  SW IV 1000c  SW IV 1000c | 0.1  10  0.1  20  0.1  30  0.1  40  0.1  50  0.1  60  0.1  70  0.1  80  0.1  90  0.1  100 | 0.286  0.147  0.096  0.610  0.132  0.478  0.934  0.659  0.135  0.199  0.622  0.163  0.262  0.061  0.800  0.153  0.174  0.947  1.000  0.720 |  | SKEL IV 100+  SKEL IV 100+  SKEL IV 200+  SKEL IV 200+  SKEL IV 300Sc  SKEL IV 300Sc  SKEL IV 400+  SKEL IV 400+  SKEL IV 500+  **SKEL IV 500+**  SKEL IV 600Sc  SKEL IV 600Sc  SKEL IV 700+  SKEL IV 700+  SKEL IV 800+  SKEL IV 800+  **SKEL IV 900**  SKEL IV 900  SKEL IV 1000Sc  SKEL IV 1000Sc | 0.1  10  0.1  20  0.1  30  0.1  40  0.1  **50**  0.1  60  0.1  70  0.1  80  **0.1**  90  0.1  100 | 0.953  0.058  0.889  0.547  0.052  0.835  0.080  0.052  0.302  **0.010**  0.869  0.078  0.181  0.143  0.062  0.368  **0.009**  0.314  0.190  0.931 |  | **AGGR III 100**  **AGGR III 100**  **AGGR III 200**  AGGR III 200  AGGR III 300  AGGR III 300  AGGR III 400  **AGGR III 400**  **AGGR III 500**  AGGR III 500  AGGR III 600b  **AGGR III 600b**  AGGR III 700  AGGR III 700  AGGR III 800  AGGR III 800  AGGR III 900  AGGR III 900  **AGGR II 1000**  AGGR II 1000 | **0.1**  **10**  **0.1**  20  0.1  30  0.1  **40**  **0.1**  50  0.1  **60**  0.1  70  0.1  80  0.1  90  **0.1**  100 | **0.008**  **0.028**  **0.021**  0.632  0.722  0.186  0.385  **0.006**  **0.000**  0.400  0.708  **0.005**  0.474  0.741  0.667  0.919  0.711  0.868  **0.032**  0.606 |

*p* values of one-sample *t*-tests of *discrete* oxygen measurements (*n* = 3) versus *continuous* oxygen measurements (*n* = 1) are shown. *p*<0.05 (bold) indicates that *discrete* and *continuous* oxygen measurements resulted in significantly different values of cumulative oxygen consumption. For AGGR experiments, cumulative oxygen consumption was normalized to aggregate volume before statistical testing.

**Table S4: Statistical comparison of relative respiration rates during the *Pressure* phase** **of SW, SKEL, and AGGR experiments calculated from *discrete* (24 h) versus *continuous* oxygen measurements (last 4 h).**

| **SW Experiment** | **Pressure (MPa)** | ***p* (a)** | ***p* (b)** |  | **SKEL Experiment** | **Pressure (MPa)** | ***p* (a)** | ***p* (b)** |  | **AGGR Experiment** | **Pressure (MPa)** | ***p* (a)** | ***p* (b)** |
| --- | --- | --- | --- | --- | --- | --- | --- | --- | --- | --- | --- | --- | --- |
| SW II 100  SW V 100  SW II 200b  SW III 200c  SW II 300  SW III 300c  SW II 400  SW V 400  SW II 500  SW II 500b  SW V 500c  SW II 600  SW III 700  SW II 800  SW III 800  SW V 800  SW III 900  SW V 900  SW II 1000  SW IV 1000c | 10  10  20  20  30  30  40  40  50  50  50  60  70  80  80  80  90  90  100  100 | 0.091  0.093  0.849  0.757  0.137  0.860  0.577  0.616  0.735  0.812  0.431  0.636  0.957  0.324  0.705  0.524  0.398  0.620  0.448  0.386 | 0.123  0.312  0.490  0.757  0.137  0.860  0.432  0.337  0.735  0.812  0.431  0.364  0.517  0.324  0.705  0.524  0.284  0.513  0.198  0.744 |  | SKEL IV 100+  SKEL IV 100S  SKEL IV 200+  SKEL IV 200S  SKEL IV 300S  SKEL IV 400+  **SKEL IV 400S**  SKEL IV 500+  SKEL IV 500SA  SKEL III 600  **SKEL IV 600Sc**  SKEL IV 600S  **SKEL IV 700+**  **SKEL IV 700S**  **SKEL IV 800**  **SKEL IV 800+**  **SKEL IV 900**  **SKEL IV 1000+**  **SKEL IV 1000S**  **SKEL IV 1000Sc** | 10  10  20  20  30  40  **40**  50  50  60  **60**  60  **70**  **70**  **80**  **80**  **90**  **100**  **100**  **100** | 0.960  0.112  0.443  0.086  **0.044**  0.197  **0.047**  0.059  **0.036**  0.518  **0.005**  0.053  **0.014**  **0.008**  **0.012**  **0.005**  **0.002**  **0.002**  **0.005**  **0.018** | **0.018**  **0.006**  0.459  **0.014**  0.858  0.197  **0.047**  0.130  0.124  0.108  **0.006**  0.065  **0.020**  **0.010**  **0.012**  **0.005**  **0.003**  **0.002**  **0.005**  **0.018** |  | AGGR III 100  AGGR III 200  AGGR III 300  AGGR III 400  AGGR III 500  AGGR III 600  **AGGR III 700**  **AGGR III 800**  **AGGR III 900**  **AGGR II 1000**  **AGGR III 1000** | 10  20  30  40  50  60  **70**  **80**  **90**  **100**  **100** | 0.385  0.201  0.061  0.680  0.133  **0.038**  **0.003**  **0.016**  **0.023**  **0.001**  **0.001** | 0.755  **0.018**  0.816  0.680  0.982  0.168  **0.005**  **0.016**  **0.028**  **0.001**  **0.001** |

*p* values of one-sample *t*-tests of *discrete* oxygen measurements (*n* = 3) versus *continuous* oxygen measurements (*n* = 2 [replicates (a) and (b)]) are shown. *p*<0.05 (bold) indicates that *discrete* and *continuous* oxygen measurements resulted in significantly different relative respiration rates. For AGGR experiments, respiration rates were normalized to aggregate volume before statistical testing.


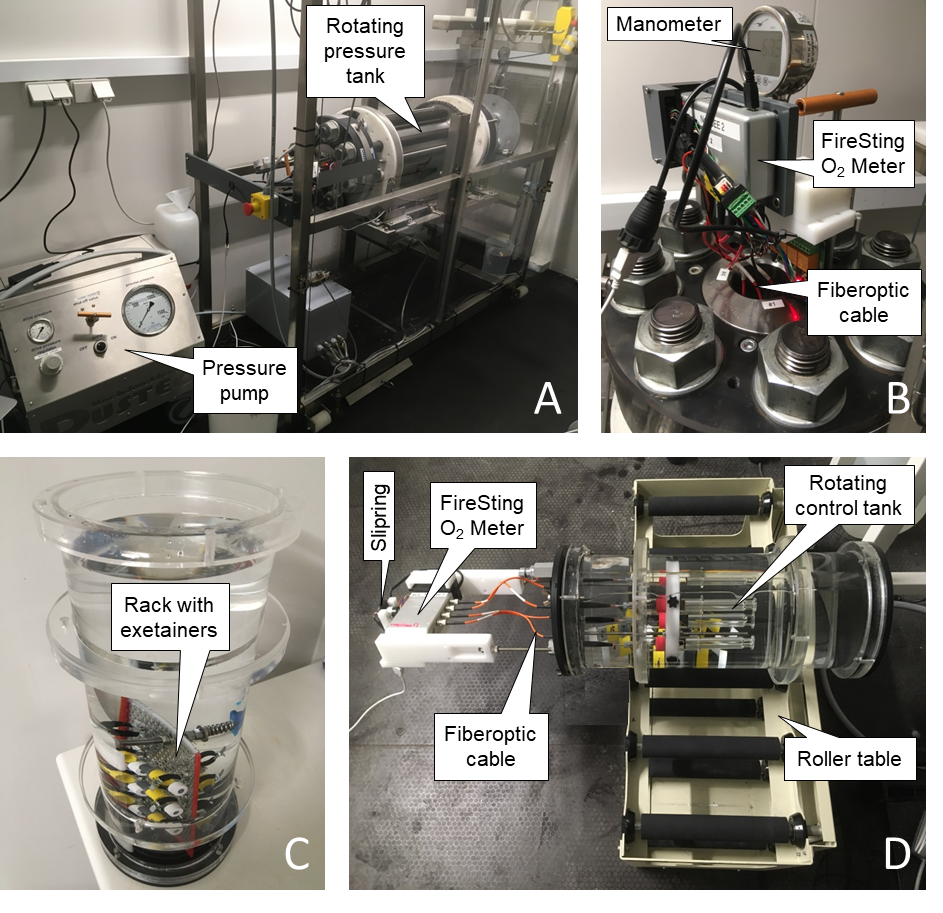


**Figure S1: Pictures of pressure and control tank.**

(A) High-pressure setup with rotating pressure tank in horizontal (operational) position within a custom-made frame for tilting the tank, (B) detail of the outer side of the lid of the pressure tank (see also **Figure S2**), (C) open, water-filled control tank with rack for sample-filled exetainers, and (D) rotating control tank in operational position on a roller table.


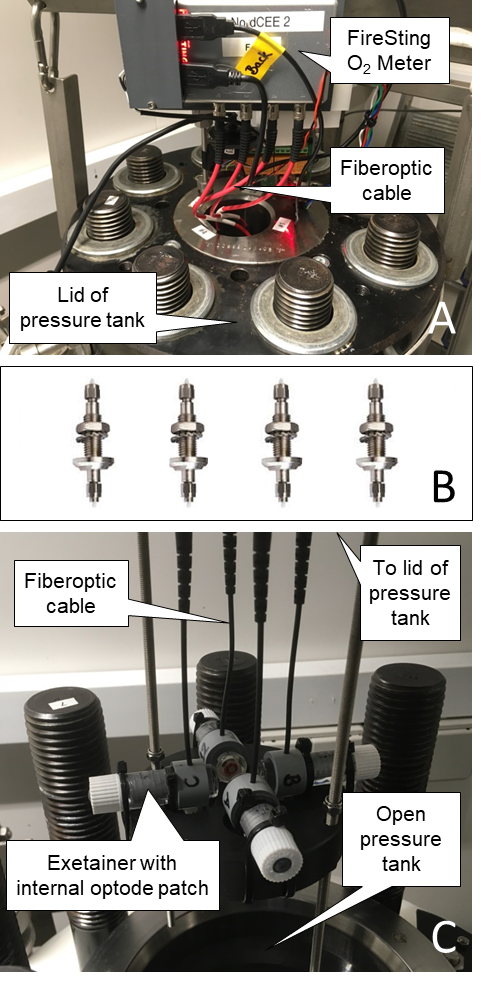


**Figure S2: Pictures of setup for optode-based, *continuous* oxygen measurements.**

(A) Outer side of the lid of the pressure tank with a 4-channel FireSting O_2_ Meter and 4 fiberoptic cables, (B) 4 high-pressure-rated fiberoptic feedthroughs mounted inside the lid of the pressure tank to connect inner and outer fiberoptic cables, and (C) 4 fiberoptic cables reaching from the inside of the lid of the pressure tank to 4 exetainers with internal optode patches for optical oxygen measurements in SW, SKEL, or AGGR samples.


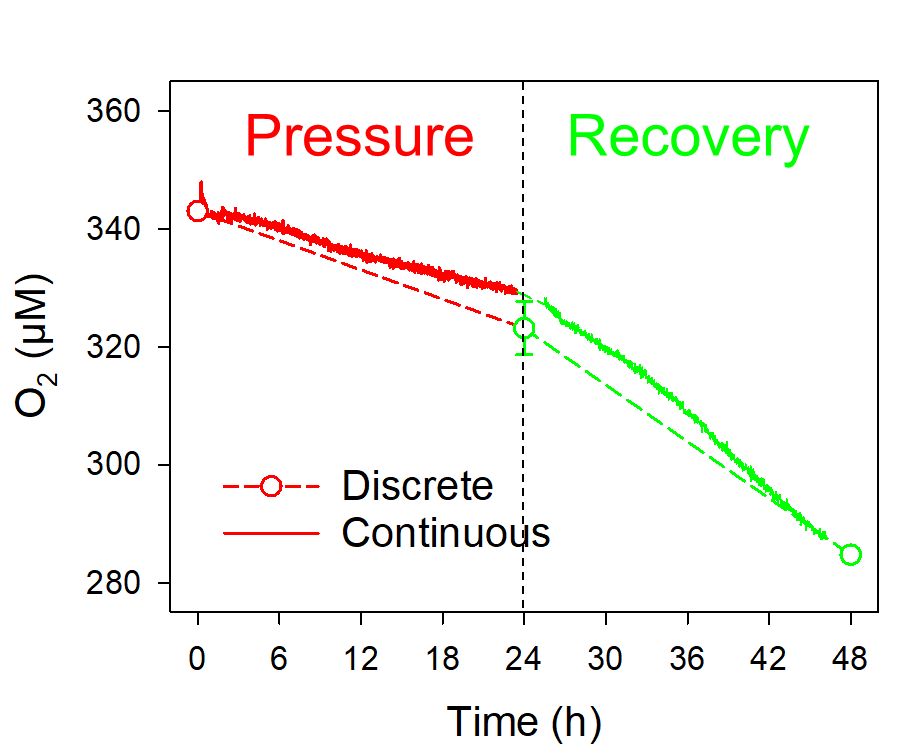


**Figure S3: Time schedule of pressure experiments.**

Each experiment was divided into two consecutive phases: (i) the *Pressure* phase during which the effect of high pressure levels/compression was tested and (ii) the *Recovery* phase during which (a) the reversibility of potential pressure effects and/or (b) the effect of decompression was tested. For each experiment, *continuous* oxygen measurements were made during the *Pressure* and *Recovery* phase, while *discrete* oxygen measurements (and other samplings) were made before and after the *Pressure* and *Recovery* phase. The same time schedule was adopted for the control incubations, while maintaining 0.1 MPa during the entire 48 h. Example oxygen time series are taken from an SW experiment at 50 MPa.

**Figure S4: Overlay of *discrete* and *continuous* oxygen measurements.**

Example oxygen time series in (A) SW, (B) SKEL, and (C) AGGR experiments at different pressure levels are shown. *Continuous* (red and green lines) and *discrete* oxygen data (blue dashed lines) were obtained from optical oxygen measurements. (B,C) Light and dark grey shading mark phases of low and high respiration activity, respectively; numbers in shaded areas give respiration rate in µM/h (SKEL) or µmol/aggregate×d (AGGR); star symbols mark linear regressions with normally distributed residuals (Shapiro-Wilk). Turquoise shading marks last 4 h of *Pressure* phase whose respiration rate was used for comparisons with *discrete* oxygen measurements (**Figure 4**). For *discrete* oxygen data, means ± standard deviation of *n* = 3-6 replicate incubations are shown. Note that aggregates differ in size between incubations.

**Figure S5: Relative growth of microbial cells in SW experiments** during the (A) *Pressure* phase and (B) *Recovery* phase. Relative growth was calculated as the ratio of increase in cell abundance at high over atmospheric pressure expressed in %. For SKEL experiments, net growth was not significantly different from zero. For AGGR experiments, net growth in control and pressure incubations could not be calculated because a data point just before the *Pressure* phase does not exist. Values of higher and lower than 100% (dashed vertical line) correspond to a relative increase and decrease in growth at high pressure, respectively. Means ± standard deviation of 3 replicates are shown.

**Figure S6: Aggregate characteristics.**

Correlations between aggregate volume and (A) carbon contents and (B) sinking speed are shown. Regression lines (black dashed lines), equations, and coefficients are given. Red dashed lines indicate the carbon content and sinking speed of an average-sized aggregate (volume = 24 mm^3^).
